# Supplementary material for: Evaluating the Necessity of Adaptive RT and the Role of Deformable Image Registration in Lung Cancer with Different Pathologic Classifications
Source: Diagnostics (Basel). 2023 Sep 15;13(18):2956. doi: 10.3390/diagnostics13182956 (PMC10527903; doi:10.3390/diagnostics13182956)
Supplement: Supplementary file 1 [file diagnostics-13-02956-s001.zip › diagnostics-2560281-supplementary.pdf]

Supplementary Methods

Patient Simulation and Treatment Planning Process

The initial treatment plan was established based on the planning kVCT image acquired using a 16-channel computed tomography simulator (Brilliance CT Big Bore; Philips Medical Systems, Cleveland, OH, USA). An image was taken with a thickness of 2 mm at 120 kVp and 50 mA using the 4D-CT technique, considering the patient's respiration. An intensity-modulated RT (IMRT) treatment plan composed of 7–9 beams using 6 MV photons was established using an RT planning system (Eclipse, 16.01.10 Platform, Varian Medical System, Palo Alto, CA, USA). The prescribed dose for the PTV was 40–44 Gy, divided into 20–22 doses of 2 Gy each, prescribed to include 95% of the PTV.

Evaluation of Treatment Plan Consistency

The verification process is based on the following key dosimetric indicators: conformity index (CI) (Equation S1), radical dose homogeneity index (rDHI) (Equation S2), and moderate dose homogeneity index (mDHI) (Equation S3). These indices reflect various aspects of RT plans and play a crucial role in evaluating the consistency of effective treatment plans for tumors. The calculation methods for each index are as follows:

$$Conformality\ IndexRTOG = VRI / TV \quad (S1)$$

where VRI is volume of reference isodose and TV is target volume;

$$rDHI = min/Dmax \quad (S2)$$

where Dmin is minimum dose and Dmax is maximum dose; and

$$mDHI = D{\geq}95\%/D{\geq}5\% \quad (S3)$$

where D≥95% implies 95% dose coverage of the target volume and D≥5% implies 5% dose

coverage of the target volume.

To set identical conditions for the tumors in each patient's initial and ART plans, any case in which the difference in all indices was >2% required reapplication of the ART plan. This process allowed the maintenance of the same conditions while verifying whether each treatment plan was effectively applied.

Data Collection and Parameter Setting

We analyzed four instances of data obtained from each patient's treatment plan and rCT. First, we tracked volume changes in the GTV for each patient. Initially, we measured the changes in the GTV from rCT1 to the three subsequent rCTs. In this manner, we monitored the changes in the GTV during the treatment process in real-time and explored the potential for treatment plan adjustments based on these changes (rCT1 vs. rCT2, rCT1 vs. rCT3, and rCT1 vs. rCT4). Second, we measured the changes in GTV between each rCT (rCT1 vs. rCT2, rCT2 vs. rCT3, and rCT3 vs. rCT4). Finally, we analyzed changes in other critical organs at risk (OAR) for each rCT using a dose-volume histogram (DVH). This analysis included the V20Gy(cm3), V30Gy (cm3), and mean dose (Dmean, Gy) of the total lung; the maximum and mean dose (Dmax and Dmean, Gy) of the esophagus; and the V20Gy, V30Gy, V40Gy, and mean dose (Dmean, Gy) of the heart. We extracted volume data in cm3 units because the imaging range of CBCT is relatively smaller than that of the initial CT, making it challenging to encapsulate the lungs or other organs entirely. Therefore, measuring the volume in cm3 is more accurate and useful for interpreting the results.
